# Between-population differences in the genetic and maternal components of body mass in roe deer

**DOI:** 10.1186/s12862-018-1154-9

**Published:** 2018-03-28

**Authors:** E. Quéméré, J. M. Gaillard, M. Galan, C. Vanpé, I. David, M. Pellerin, P. Kjellander, A. J. M. Hewison, J. M. Pemberton

**Affiliations:** 1 CEFS, INRA, Université de Toulouse, Castanet-Tolosan Cedex, F-31326 France; 2 Université Lyon 1, CNRS, UMR 5558, Laboratoire de Biométrie et Biologie Evolutive, F-69622 Villeurbanne, France; 3 CBGP, INRA, CIRAD, IRD, Montpellier SupAgro, Univ. Montpellier, F-34988, Montferrier-sur-Lez Cedex, France; 4 GenPhySE, INRA, Université de Toulouse, ENVT, Castanet-Tolosan, F-31326 France; 5 ONCFS, DER, UR Cervidés-Sanglier, Paris, France; 6 Grimsö Wildlife Research Station, Department of Ecology, Swedish University of Agricultural Sciences, SE-730 91 Riddarhyttan, Sweden; 7 Institute of Evolutionary Biology, School of Biological Sciences, University of Edinburgh, Edinburgh, EH9 3FL UK

**Keywords:** Additive genetic variance, Animal model, Heritability, Mammals, Ungulates, *Capreolus capreolus*

## Abstract

**Background:**

Understanding the genetic and environmental mechanisms governing variation in morphology or phenology in wild populations is currently an important challenge. While there is a general consensus that selection is stronger under stressful conditions, it remains unclear whether the evolutionary potential of traits should increase or decrease with increasingly stressful conditions. Here, we investigate how contrasting environmental conditions during growth may affect the maternal and genetic components of body mass in roe deer, the most abundant and widespread wild ungulate in Western Europe. Body mass is a key life history trait that strongly influences both survival and reproductive performance in large herbivores. We used pedigrees and animal models to determine the variance components of juvenile and adult winter body mass in two populations experiencing contrasting early-life conditions.

**Results:**

Our analyses showed that roe deer at Chizé, where habitat was poor and unpredictable, exhibited very low genetic variance in juvenile body mass. Instead, variance in mass was mainly driven by among-cohort differences in early-life conditions and maternal environment. In contrast, roe deer at Bogesund, where resource availability during the critical period of fawn rearing was higher, displayed a substantial level of genetic variance in body mass. We discuss the potential role of past demography and viability selection on fawn body mass on the erosion of genetic variance in the poor habitat.

**Conclusions:**

Our study highlights the importance of accounting for both spatial (i.e. between-population variation) and temporal (i.e. cohort variation) heterogeneity in environmental conditions, especially in early life, to understand the potential for adaptive responses of wild populations to selection.

**Electronic supplementary material:**

The online version of this article (10.1186/s12862-018-1154-9) contains supplementary material, which is available to authorized users.

## Background

Understanding the environmental and evolutionary mechanisms governing variation in phenology [1, 2], behavior [3] and morphology [4, 5] is an important challenge in evolutionary ecology [6, 7]. Phenotypic plasticity, the ability of a given genotype to express different phenotypes depending on environmental conditions, may enable individuals to cope with environmental changes in the short term, but a micro-evolutionary (genetic) response may be required to sustain a directional response over longer periods [8]. The evolutionary potential of a trait is traditionally determined by its narrow-sense heritability (*h*^*2*^), which is defined as the fraction of the total phenotypic variance (V_P_) due to the additive effects of genes, measured as the additive genetic variance (V_A_) [9]. Therefore, predicting the ability of a trait to respond to natural selection requires knowledge about the amount of genetic variation for that trait, but also about all environmental factors, including maternal effects, that may affect its expression during ontogeny.

It is widely recognized that both selection and the expression of quantitative genetic variation can vary across environmental conditions [10]. However, while there is a general consensus that selection is stronger under harsh conditions [11], it remains unclear whether genetic variation should increase or decrease with increasingly stressful conditions [12–15]. In a recent meta-analysis, Rowinski & Rogell [16] pointed out that highly stressful conditions generally lead to higher genetic variance in life-history traits. However, a large proportion of the studies analyzed in this work were conducted on *Drosophila* spp. in laboratory conditions where the nature and intensity of induced stress and the evolutionary mechanisms implicated (e.g. increased rates of mutation/recombination, expression of new genes) are different from those observed in natural environments [13, 17]. By contrast, most studies in wild bird or mammal populations ([12, 14, 18, 19], but see [20]) have provided evidence for higher heritability under favourable conditions, although differences in *h*^*2*^ often arise from differences in the levels of environmental or residual variance rather than from different levels of V_A_ per se. More recently, Martinez-Padilla et al. [21] found a strong inverse relationship between environmental harshness and evolutionary potential of morphological traits across multiple bird populations covering a wide range of environmental conditions. This lack of consensus in the literature highlights the need for further empirical studies on a greater diversity of taxa to understand how environmental heterogeneity in the wild may affect the expression of genetic variance and, thus, the potential for evolution of fitness-related traits.

In a similar manner, little is known about how variation in environmental conditions may alter trait variation linked to maternal effects. Maternal effects are a special case of environmental effects that occur when a mother influences her offspring’s phenotype independently of the offspring’s genetic make-up. These effects are expected to be prevalent for traits expressed during early life stages, especially in taxa that provide substantial maternal care [22]. Variation in offspring growth may be partly shaped by among-mother differences in the quantity or quality of milk provided. If milk production is influenced by food availability for mothers, this should give rise to maternal environmental effects. However, maternal effects may also result from among-mother genetic variation (maternal genetic effects) and may evolve in response to selection acting on offspring traits [23]. Whether genetic or environmental, maternal effects are expected to vary in intensity in relation to the environment experienced by the mother during the offspring birth and rearing periods [24, 25]. Again, the environmental dependence of maternal effects has been little studied in wild populations to date, and there is currently no consensus about the expected direction of differences in the expression of maternal effects as a function of environmental harshness [13].

In this study we aim to investigate how variation in environmental conditions during early growth may affect the maternal and genetic components of body mass in the roe deer (*Capreolus capreolus,* Linnaeus 1758), the most abundant and widespread large herbivore in the wild in Western Europe [26]. Body mass has been intensively studied in a wide range of vertebrates because of its marked influence on both survival and reproductive performance [27, 28] and, more generally, population dynamics [29–31]. We analyzed body mass data collected from two long-term individual-based studies of populations living in very different ecological contexts. The roe deer population at Chizé, in Western France, experiences large inter-annual variation in habitat quality, especially during the critical fawn rearing period in spring and summer [32]. By contrast, roe deer at Bogesund, in South-eastern Sweden, have access to rich and rather more predictable resources during early life.

We investigated how variance components of juvenile body mass were associated with yearly variation in early-life conditions at Chizé where substantial cohort effects have previously been documented [33]. Roe deer are income breeders (sensu Jönsson [34]) that rely on current resource intake to offset the costs of reproduction [35]. Previous work has shown that population density and spring temperature during early life markedly affect fawn early growth and, thereby, juvenile winter body mass in roe deer [33, 36, 37]. We thus expected a greater influence of early-life environmental conditions (i.e. cohort variation) on juvenile body mass at Chizé than at Bogesund, with carry-over effects on adult body mass [38]. The lack of consensus among previous studies prevented us from formulating explicit predictions regarding the direction of any differences in the expression of the additive genetic variance, maternal variation and heritability in relation to environmental conditions, whether between the two populations or among years at Chizé.

## Methods

### Study sites and data collection

The study used data collected from populations of roe deer that differ markedly in terms of environmental conditions. The first population inhabits a 2614 ha enclosed forest in western France, the Réserve Biologique Intégrale of Chizé (GPS coordinates: 46°11’N;-0°34’W). Chizé has an oceanic climate with some Mediterranean influence, with mild winters and warm, often dry, summers. The primary production of this forest is quite low due to poor soils, and the availability of food for roe deer is limited by summer drought. Previous work has shown that the demographic performance of roe deer in Chizé is strongly limited by a combination of harsh climatic events and a relatively high population density in certain years [39] which depresses both juvenile body mass [36] and fawn survival [32]. The second study site, Bogesund, is a small peninsula (2600 ha) in south eastern Sweden (GPS coordinates: 59°38’N; 18°28′E) surrounded by water on all sides except to the north. The landscape is composed of a mosaic of forested (65% of the surface area) and field habitats. The climate in Bogesund is more seasonal than in Chizé, with snow cover that partially limits access to food for deer during 1–3 winter months. However, during the critical period for fawn rearing, in spring and summer, the climate is mild, with more precipitation than at Chizé, so that roe deer have access to richer and more predictable resources (see [40] for further comparisons between Scandinavia and France in terms of seasonality and stochasticity of resource availability). Neither summer nor winter climate influenced winter fawn body mass in this population [37].

The two populations have been intensively monitored for more than 30 years using annual capture-mark-recapture sessions (see [41, 42] for further details). A substantial proportion of individuals were first captured as neonates during spring (within their first three weeks of life). Then, each year, during winter, more than 50% of individuals were caught either as juveniles (i.e. at about 8 months old) or as adults (i.e. at 20 months old or older) in box traps (Bogesund) or nets (Chizé). At each capture, all animals are sexed, weighed, measured (hind foot length), and inspected for marks or newly marked with ear-tags and collars (during winter captures only). Age is either known (for individuals first caught as newborns or juveniles) based on tooth eruption [43] or estimated from tooth wear (for individuals older than 1 year of age). As the reliability of age assessment from tooth wear is rather low [44], we pooled all animals older than one year into an adult age class and we took the median adult body mass when the same individual was repeatedly weighed during its adult life. As juvenile body mass at the onset of winter changes with Julian date of capture at both Chizé (slope of the linear regression between body mass and Julian date ± SE = 0.012 ± 0.002, *t* = 7.09, *P* < 10^− 3^) and Bogesund (− 0.0047 ± 0.002, *t* = 2.21, *P* = 0.028) [37], juvenile body mass was standardized for capture date prior to analysis by adjusting juvenile body mass to February 14, the median date of the entire capture period in both sites. Since 1996 (at Chizé) and 1988 (at Bogesund), ear punches have been collected for genetic analysis from individuals at their first capture. Some tissue samples and body mass measurements were also obtained from individuals that were either shot or removed during experimental manipulation of density in the two study areas (the removal or hunting of roe deer was not related to body mass; for further details see [41, 45]).

#### Pedigree reconstruction

A total of 1941 and 2109 roe deer were genotyped using overlapping sets of 11 and 21 microsatellites at Chizé and Bogesund, respectively (see [46] for the detailed genotyping protocol). Note that we used twice as many loci in Bogesund than in Chizé because the level of polymorphism was much lower at Bogesund (see results). We reconstructed a multigenerational pedigree for both populations to quantify relatedness among all pairs of individuals. Maternity was either known from field observations or assigned using parentage analysis as implemented in COLONY 2 [47, 48], while paternity was inferred solely from COLONY 2 (see supplementary material). The pedigree for Bogesund contained 2066 individuals (born between 1983 and 2011), with 1041 maternal links (50%) and 789 paternal links (39%) (from 325 different dams and 381 different sires). The pedigree for Chizé contained 1696 individuals (born between 1996 and 2012), with 675 maternal links (48%) and 645 paternal links (46%) (from 360 different dams and 304 different sires).

#### Estimation of variance components

We investigated how the variance components of juvenile and adult body mass differed between populations. As a first step, we sought to tease apart the relative importance of the early-life environment, the mother (both genetic and permanent environment effects) and genes on juvenile body mass. For each population, we fitted a univariate model which partitions the total phenotypic variance (V_P_) in juvenile body mass (JBM) into additive genetic variance (V_A_), early life environmental variance represented by birth year (V_BY_), maternal environmental variance (V_ME_), maternal additive genetic variance (V_MA_), and residual variance (V_R_). Maternal identity was thus fitted twice, once including pedigree information (i.e. genetic relatedness among mothers) and once without, to partition maternal effects into additive genetic (MA) and permanent environmental (ME) effects. To ensure model convergence, we assumed no covariance between direct and maternal additive genetic effects. Variance components were estimated using the restricted maximum likelihood (REML) method [49] in ASReml 3.0 [50]. Sex was included as a fixed effect (two-level factor) because the average body mass differs between males and females [51]. Statistical significance of random components was assessed using likelihood ratio tests (LRT). Narrow-sense heritability *h*^2^ was calculated as the ratio of additive genetic variance (V_A_) to total phenotypic variance (V_P_). The maternal genetic effect was calculated as the ratio of V_MA_ to total phenotypic variance (V_P_) and total heritability *h*^*2*^_*T*_ as (V_A_ + 0.5V_MA_)/V_P_ following Willham [52]. Because body mass increases with age and differs between populations (see below), scale effects may preclude direct comparison of the magnitude of variance components between populations and between age-classes. For each variance component, we therefore calculated the corresponding coefficient of variation (CV) [53] by scaling the variance relative to the mean as follows: CV_i_ = 100 √V_i_/ $\overline{\mathrm{X}}$_i_ with the variance of the component (*i* = A, BY, ME, MA, R or (P = phenotypic)) and $\overline{\mathrm{X}}$_i_ the mean of the trait. To explicitly test whether variance components for juvenile body mass differed between Chizé and Bogesund, we combined the phenotypic data set and pedigree information from both populations [20] and fitted bivariate models in which mass in the two populations was considered as two different traits (i.e. JBM_CH_ and JBM_BO_). For each random component, we fitted a first model constraining the respective variance components to be equal in each population. We then used LRTs to compare this model and a bivariate model in which the variance components were allowed to differ between populations. Differences between coefficients of variation were tested by t-tests (assuming Gaussian uncorrelated estimates) using the “BSDA” R package (Arnholt 2012).

In a second step, to investigate how variance components of body mass differed between age classes (i.e. juvenile vs. adult), we next fitted a bivariate animal model for each population with variance decomposition for mass in each age class. We used the same variance partitioning as in the univariate models described above except that, to ensure model convergence, we fitted a simple maternal effect without distinguishing a maternal genetic effect. Genetic, maternal and environmental covariances between juvenile and adult body masses and corresponding between-age correlations (r_G_, r_M,_ r_BY_) were also estimated. To test between-age differences in variance components in the two populations formally, we used the same procedure as described above for between-population comparisons (i.e. LRTs between “relaxed” and “constrained” variance components).

#### Influence of early conditions on variance components of juvenile body mass

For juvenile body mass at Chizé, we analyzed the sensitivity of the maternal variance component (i.e. the only statistically significant source of variance in juvenile body mass– see results) to yearly fluctuations in early environmental conditions using the analytical technique of “random regression models” *[*sensu [54] *]*. We used the following model: *JBM*_*ijky*_ = *μ* + *Sex*_*k*_ + *year*_*y*_ + *a*_*i*_ + *m*_0*j*_ + *m*_1*j*_*E*_*y*_ + *e*_*ijky*_ where *JBM*_*ijky*_ is the juvenile body mass of animal i born in year y, of sexe k, from mother j. The sex (*Sex*_*k*_) and the birth year (*year*_y_) were fitted as fixed effects (two and thirty levels respectively). *a*_*i*_ is the direct genetic effects of animal i (with a mean of zero and variance AV_A_), *m*_0*j*_ and *m*_1*j*_ the 2 random regression coefficients of mother j (i.e. intercept and slope, with a mean of zero and a covariance- matrix $$ \left[\begin{array}{cc}{\sigma}_{m_0}^2& {\sigma}_{m_0{m}_1}\\ {}{\sigma}_{m_0{m}_1}& {\sigma}_{m_1}^2\end{array}\right] $$)*, e*_*ijky*_ is the residual term (with a mean of zero and variance V_R_). A is the known relationship matrix. *E*_*y*_ *is the* environmental quality during the birth year *y* and was measured as the cohort-specific juvenile survival over the spring-summer, i.e. the proportion of juveniles surviving to 8 months of age. The variances (and standard errors) of maternal effect in juvenile environment *E*_*y*_ were computed using the following formula in ASReml 3.0: ${\sigma}_{m_0}^2+2{E}_y{\sigma}_{m_0{m}_1}+{E}_y^2{\sigma}_{m_1}^2$. A previous study in this population has shown that neonatal survival decreases with increasing density, and increases with increasing rainfall in May and June [33]. V_A_ and V_R_ were assumed to be constant across environments. To test this assumption, we ran bivariate animal models using data subsets for good and poor environments (based on upper and lower 50 percentiles of E). We did not find any detectable differences in estimated additive genetic (χ^2^ = 2.41, d.f. = 1, *P* = 0.12) or residual (χ^2^ = 0.37, d.f. = 1, *P* = 0.79) variances between the two environments.

## Results

### Comparison of variance components of juvenile body mass among populations

There was a marked difference in average body mass of roe deer between the two populations for both juveniles (F_1,1272_ = 178, *P* < 10^− 4^) and adults (F_1,1195_ = 291, *P* < 10^− 4^) (Table 1). At any given age, and in both sexes, roe deer were heavier at Bogesund than at Chizé. The univariate analysis of variance components of juvenile winter body mass also revealed marked differences between populations (Table 1). At Bogesund, standardized additive genetic variance (CV_A_) among juveniles (11.34 ± 2.14) (mean ± SE) was three times larger than at Chizé (3.85 ± 2.87). In the direct test for differences in V_A_ between Chizé and Bogesund, V_A_ differed between populations (*χ*^*2*^ = 4.5, d.f. = 1, *P* = 0.03). Early-life conditions (V_BY_) had a pronounced effect on juvenile body mass in both populations, but this effect tended to be larger at Chizé (V_BY_ = 2.67 ± 1.07) than at Bogesund (1.3 ± 0.52) (*χ*^*2*^ = 1.8, d.f. = 1, *P* = 0.17). The same trend was observed when considering CV_BY_ (11.45 ± 2 .14 at Chizé versus 7.98 ± 1.59 at Bogesund, t_1270_ = 1.16, *P* = 0.24). We also detected a maternal effect in both populations, but this derived from different sources: at Chizé, the maternal effect was almost entirely due to among-mother environmental differences (CV_ME_ = 6.11 ± 1.07 and CV_MA_ = 0.02 ± 0.01), whereas at Bogesund, we only detected maternal genetic variance (CV_ME_ = 1.12 ± 17.03 and CV_MA_ = 8.29 ± 3.07). We found substantial direct heritability (h^2^) of juvenile winter body mass at Bogesund (h^2^ = 0.44 ± 0.11), while it was not statistically different from zero at Chizé (h^2^ = 0.05 ± 0.07, *χ*^*2*^ = 0.46, d.f. = 0.5, *P* = 0.24). Total heritability, incorporating both maternal and direct genetic effects (h_T_^2^), was 0.04 ± 0.07 at Chizé and 0.56 ± 0.14 at Bogesund.

**Table 1:** Variance components from univariate models of juvenile body mass in the two study populations

Population	CHIZÉ	BOGESUND
Mean JBM	14.27(0.09)	16.27(0.11)
N_JUV_	782	490
N_MOT_	572	309
V_P_	6.68(1.02)	5.97(0.68)
V_A_	0.30(0.45)	2.63(0.97)
V_BY_	2.67(1.01)	1.3(0.52)
V_ME_	0.76(0.27)	0.03(0.78)
V_MA_	< 10^−5^	1.4(1.04)
V_R_	2.94(0.4)	0.62(0.64)
h^2^	0.05(0.07)	0.44(0.16)
by^2^	0.4(0.09)	0.22(0.07)
me^2^	0.11(0.04)	0
ma^2^	0	0.23(0.17)
r^2^	0.44(0.08)	0.1(0.11)
CV_P_	18.08(1.38)	17.11(0.97)
CV_A_	3.85(2.87)	11.34(2.08)
CV_BY_	11.45(2.14)	7.98(1.59)
CV_ME_	6.11(1.07)	1.12(17.03)
CV_MA_	0.02(0.01)	8.29(3.07)
CV_R_	12(0.82)	5.52(2.85)
h_T_^2^	0.05(0.07)	0.56(0.14)

JBM: winter juvenile body mass in kg; N_JUV_: number of juveniles used in the study; N_MOT:_ number of mothers used in the study; Components of phenotypic variance V_P_ (V_A_: additive genetic variance; V_BY_: early environment variance; V_ME_: maternal environment variance V_MA_: maternal additive genetic variance, V_R_: residual variance) and their associated ratios (h^2^: heritability, me^2^, ma^2^, by^2^ and r^2^) and coefficients of variation (CV_A_, CV_ME_, CV_MA_, CV_BY_, CV_R_, CV_P_) (see main text for details). h_T_^2^: total heritability. All values (except sample sizes) are given as the mean with standard errors indicated in brackets

### Comparison of variance components of body mass across age classes

**Fig. 1:**
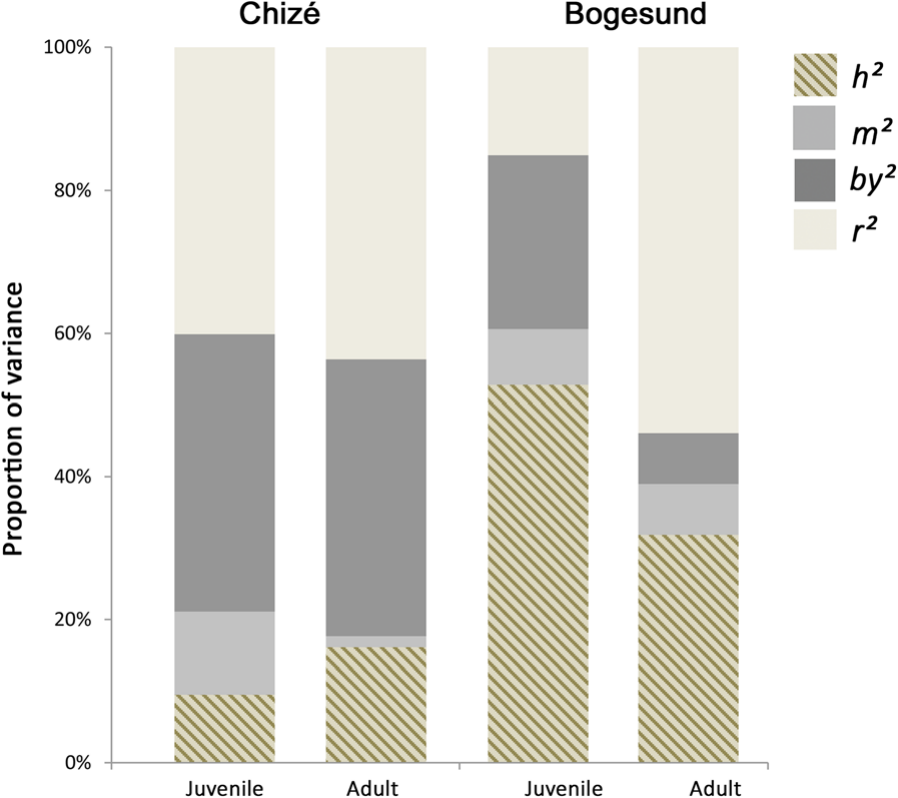
Age-class-dependent differences in variance components for roe deer winter body mass at Chizé and Bogesund derived from a bivariate model for each population. Proportion of the phenotypic variance of juvenile and adult body mass explained by heritability (h^2^), maternal effect (m^2^), birth year effect (by^2^), and residual effect (r^2^)

Bivariate models produced similar estimates for variance components in juvenile body mass as the univariate model (Table 2, Fig. 1). At Chizé, the bivariate analysis revealed a low but statistically significant additive genetic variance of body mass in adults (V_A_ = 1.33 ± 0.45) (Table 2). V_A_ tended to be higher among adults than among juveniles (V_A_ = 0.63 ± 0.36) (*χ*^*2*^ = 1.62, d.f. =1, *P* = 0.20), but this pattern was not reflected in the coefficients of variation (CV_A_) and is, therefore, due to scale effects (Table 2). At Bogesund, V_A_ was very similar among juveniles (V_A_ = 3.25 ± 0.66) and adults (V_A_ = 3.21 ± 1.13). We found substantial direct heritability of body mass among both juveniles (h^2^ = 0.53 ± 0.10) and adults (h^2^ = 0.32 ± 0.10) in Bogesund (Fig. 1). Conversely, at Chizé, direct heritability was detectable among adults only and was relatively low (h^2^ = 0.16 ± 0.06). We detected a high positive additive genetic correlation between juvenile and adult body mass at Bogesund (r_A_ = 0.66 ± 0.13) (not estimated at Chizé due the very low V_A_ in juvenile body mass).

**Table 2:** Variance components of juvenile and adult winter body mass in the two study populations derived from a bivariate model for each population

Population	CHIZÉ		BOGESUND	
Age-class	Juvenile	Adult	Juvenile	Adult
Mean BM	14.27(0.09)	22.04(0.08)	16.27(0.11)	24.09(0.09)
V_P_	6.64(0.98)	8.25(1.21)	6.15(0.67)	10.39(0.7)
N	1455		1413	
N_m_	1812		1651	
V_A_	0.63(0.36)	1.33(0.45)	3.25(0.66)	3.32(1.13)
V_by_	2.67(0.33)	3.6(0.39)	1.5(0.56)	0.75(0.36)
V_M_	0.77(0.24)	0.13(0.21)	0.48(0.34)	0.74(0.53)
V_r_	2.57(0.95)	3.19(1.18)	0.93(0.44)	5.59(0.97)
h^2^	0.1(0.06)	0.16(0.06)	0.53(0.1)	0.32(0.1)
by^2^	0.39(0.09)	0.39(0.09)	0.24(0.08)	0.07(0.03)
m^2^	0.12(0.04)	0.02(0.03)	0.08(0.06)	0.07(0.05)
r^2^	0.4(0.07)	0.44(0.08)	0.15(0.07)	0.54(0.1)
CV_P_	18.04(1.33)	13.01(0.96)	15.23(0.82)	13.38(0.45)
CV_A_	5.58(1.59)	5.23(0.89)	11.07(1.12)	7.48(0.76)
CV_BY_	11.21(2.07)	8.09(1.49)	7.51(1.4)	3.57(1.27)
CV_M_	6.15(0.96)	1.61(1.32)	4.26(1.58)	3.58(1.62)
CV_R_	11.44(0.7)	8.59(0.46)	5.91(1.4)	9.81(0.85)

BM: winter body mass in kg; N: total sample size for juveniles and adults; N_m:_ total number of measurements for juveniles and adults; components of phenotypic variance V_P_ (V_a_: additive genetic variance; V_M_: maternal variance; V_B_: early environment variance; V_R_: residual variance) and their associated ratios (h^2^: heritability, m^2^, by^2^ and r^2^) and coefficients of variation (CV_A_, CV_M_, CV_BY_, CV_R_, CV_P_). All values (except sample sizes) are given as the mean with standard errors indicated in brackets

In Bogesund, early-life environment accounted for twice as much variance in body mass of juveniles (V_BY_ = 1.5 ± 0.56) as of adults (0.75 ± 0.36) (*χ*^*2*^ = 1.52, d.f. = 1, *P* = 0.23) and this trend was reflected in the coefficients of variation (CV_BY_ = 7.51 ± 1.40 in adults vs 3.57 ± 1.27 in juveniles) (t_1549_ = 1.82, *P* < 0.07). Early-life environment accounted for about 25% (± 8%) of V_P_ in body mass among juveniles, but only about 7% (± 3%) among adults. In contrast, in Chizé, early-life environment effects were similarly high in adults (V_BY_ = 3.6 ± 0.39) and juveniles (2.67 ± 0.33) (*χ*^*2*^ = 0.74, d.f. = 1, *P* = 0.38), accounting for 40% (± 8%) of V_P_ in both age classes. Lastly, in both populations, we observed a trend for lower maternal variance in body mass of adults compared to juveniles, although this difference was only statistically significant in Chizé (*χ*^*2*^ = 4.02, d.f. = 1, *P* = 0.04). This pattern was consistent with the coefficients of variation (CV_ME_ = 6.15 ± 0.95 in juveniles vs 1.61 ± 1.32 in adults, t_2026_ = 2.79, *P* < 0.01).

### Maternal-by-environment interaction for juvenile body mass at Chizé

**Fig. 2:**
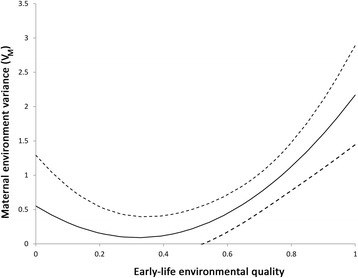
Estimated maternal variance of juvenile winter body mass in relation to early-life environmental conditions at Chizé. Early-life environmental quality was defined as the cohort-specific juvenile summer survival rate, i.e. the proportion of juveniles born that survived to 8 months of age. Dashed lines indicate standard error interval for the estimated maternal variance (V_M_). The random regression model suggests a general increase in V_M_ as early-life environment quality increases. The univariate analysis presented in Table 1 suggests that maternal variance is nearly all environmental in origin in this population

The model incorporating covariation between the maternal environmental variance component and early-life environmental conditions provided a better fit than the baseline model where variance is assumed to be constant (*χ*^*2*^ = 5.88, d.f. = 2, *P* = 0.048). We observed an increase in maternal environmental variance (V_ME_) with more favourable conditions during early life (Fig. 2). A maternal environmental effect on juvenile body mass was not detectable when environmental conditions were poor, but accounted for more than 25% of the total phenotypic variance when conditions were favourable (m^2^ = 0.25–0.41 in years when summer juvenile survival was > 0.7). From this model, the additive genetic variance and residual components of variance of body mass were estimated as V_A_ = 0.40 ± 0.46 and V_R_ = 2.79 ± 0.41, respectively.

## Discussion

Our results confirm that the quantitative architecture of body mass can differ widely among populations within a given species. Heritability of juvenile winter body mass was particularly low at Chizé due to low additive genetic variance combined with marked effects of early-life conditions, giving rise to pronounced among-cohort variation in body mass. In contrast, at Bogesund, additive genetic variance and heritability (h^2^ = 0.44) of juvenile body mass were much higher than at Chizé and concordant with the few published estimates of the heritability of juvenile mass in wild ungulates (e.g. h^2^ = 0.43 in Bighorn sheep [55], 0.58–0.64 in white-tailed deer *Odocoileus virginianus* [56]), but larger than those recently estimated in Soay Sheep (h^2^ = 0.16–0.21) [57].

### A marked long-lasting influence of early-life conditions on heritability of body mass

As predicted, among-cohort variation in early-life environmental conditions had a marked influence on juvenile winter body mass at Chizé (CV_BY_ = 11.21 ± 2.07), constituting nearly 40% of the total phenotypic variance. Since female roe deer rely almost exclusively on available resources for their high energetic needs during fawn rearing [35], early fawn growth is particularly sensitive to variations in climate conditions that occurred at Chizé, influencing both resource quantity and quantity [58]. Such high environmental variance in morphological traits has been observed under stressful conditions for many species (see [13]). However, in most cases, this resulted from spatial rather than temporal variation in resource quality (but see [14, 59] on Soay sheep). Interestingly, we found that among-cohort variation in early-life conditions remained a major driver of phenotypic variance in body mass of adults at Chizé (CV_BY_ = 8.09 ± 1.49, 39% of V_P_) in agreement with previous work [38, 39]. This carry-over effect of early-life environment meant that heritability of adult body mass was unusually low (h^2^ = 0.16 ± 0.06). At Bogesund, we observed lower, but detectable, environmental variance in juvenile body mass (CV_BY_ = 7.51 ± 1.4, 24% of V_P_) that likely reflects among-cohort variation in first winter snow conditions (e.g. > 28 days with > 10 cm of snow in 1991 vs. 5 days in 2007–2009). In contrast to Chizé, this among-cohort effect was no longer detectable at the adult stage (7% of V_P_), when the residual variance of body mass was markedly higher (54% of V_P_).

### Understanding the particularly low V_A_ in juvenile body mass at Chizé

A particularly striking result of the present study is the unusually low genetic variance in juvenile body mass at Chizé (CV_A_ = 3.85 ± 2.87) compared to Bogesund (CV_A_ = 11.3 ± 2.08). However, one must be cautious when interpreting this difference in relation to prevailing environmental conditions because our work is based on the comparison of only two populations. Such differences could, in theory, have arisen solely due to distinct past demographic histories and effective population sizes. Indeed, genetic variance is predicted to decrease with decreasing population size at the same rate as neutral variation [60]. Here, data on selectively neutral genetic markers did not provide any support for a role of recent population history because microsatellite genetic diversity was much lower in Bogesund where heritability estimates were highest (mean number of alleles per locus = 4.19 at Bogesund vs. 6.67 at Chizé). However, the two populations are thousands of kilometers away from each other and we cannot rule out a role of historical biogeographic events. An alternative hypothesis is that genetic variance of body mass at Chizé has been depleted by persistent and strong viability selection on this trait [10]. Both summer and winter juvenile body mass have a marked influence on juvenile survival at Chizé [31, 42, 61], with delayed and long-lasting effects on the reproductive performance of adults [62]. To investigate this hypothesis, we quantified the response of early fawn survival to neonatal body mass in the two populations (see supplementary material). We found strong evidence of viability selection before the first winter at Chizé (mean coefficient ± SE = 0.46 ± 0.15, *P* < 0.001) but not at Bogesund (mean coefficient ± SE = − 0.23 ± 0.19, *P* = 0.22) (Additional file 1: Figure S1). By filtering out small individuals, condition-dependent survival over the first summer at Chizé might have led to the depletion of the genetic variance of body mass after the critical stage compared to its initial level at birth [22, 63]. As the critical stage for roe deer is pre-weaning survival between May and September, body mass at 8 months of age (JBM) might reflect this process. Conversely, the much higher level of genetic variance in body mass at Bogesund may be related to the absence of detectable viability selection on juvenile body mass in this population. As food is abundant and more predictable during the critical period of fawn rearing at this site, fawn mortality by starvation is unlikely, so that survival is not related to early mass [64]. However, while appealing, this hypothesis was, for now, somewhat speculative given that previous published work has indicated that strong and persistent selection generally fails to deplete genetic variance in life-history traits that are closely related to fitness [65]. Furthermore, this hypothesis implies a strong genetic correlation between neonatal and first winter body mass and that early viability selection has a genetic component and is not entirely due to variation in micro-environmental quality [66, 67]. Further investigation is clearly needed to assess the potential role of viability selection on the depletion of genetic variance.

### Maternal genetic and environmental effects on juvenile body mass

Maternal effects arise because of variation among mothers in traits that influence offspring phenotype [23, 68]. While maternal effects have been widely quantified in diverse taxa [25, 69, 70], the presence of heritable components in maternal care and their indirect effects on offspring traits have rarely been investigated in wild populations (but see Wilson et al. [71] in Soay sheep; Räsänen et al. [72] in frogs; McFarlane et al. [73] in North American red squirrels *Tamiasciurus hudsonicus*). In some wild mammal populations, such as Soay sheep or North American red squirrels, the maternal genetic effect is the main source of heritable variation in traits that are expressed early in life, such as birth mass [71, 74]. In our study, we were unable to detect any maternal genetic variance (V_MA_) for juvenile body mass at Chizé, while this effect represented more than 20% of the total phenotypic variance at Bogesund. In mammals, maternal care is positively associated with early growth and survival of offspring [75, 76]. We can therefore hypothesize that viability selection acting on juveniles at Chizé has also eroded the among-mother genetic variation in traits that indirectly influence offspring phenotype. At Chizé, maternal effects appeared to be almost entirely generated by among-mother environmental differences and may result from long-lasting effects of early-life conditions on maternal traits. Indeed, since 40% of the variation in adult body mass is due to cohort effects, we can reasonably assume that other traits involved in maternal care are also strongly determined by the early-life conditions a given mother experiences. Environmental maternal effects could also be partly explained by pronounced spatial variation in food resources [58]. Indeed, fine-scale spatial heterogeneity in forage availability and quality markedly impacts variation in offspring growth and survival among mothers in this population.

Interestingly, our results at Chizé revealed that the magnitude of the maternal environment effect on winter juvenile body mass varies among cohorts depending on early-life climatic conditions. The effects of the maternal environment were not detectable for those cohorts that had experienced poor environmental conditions during early-life, but these effects were marked when early-life conditions were better. At Chizé, harsh climatic conditions, such as intense summer droughts, affect all individuals and likely override among-mother differences in rearing performance, leading to the suppression of maternal variance in juvenile body mass. Among-individual differences in the expression of maternal effects in relation to environmental stress have been reported in a number of wild populations [24, 25]. In blue tits (*Cyanistes caeruleus*, Linnaeus 1758), environmental stress linked to parasitism leads to an increase in variance of offspring growth rates due to shared environmental effects between nests [which is mainly determined by parental effects, 19]. In Soay sheep, another species in which females allocate a lot per breeding attempt, the maternal genetic component of variance in lamb birth mass was lower in poor environments [14].

## Conclusions

This study illustrates that the genetic, maternal and environmental architecture of a given trait may vary strongly among wild populations of the same species. A striking pattern is the very low level of additive genetic variance in juvenile body mass we reported at Chizé, which suggests that this trait cannot evolve in response to the direct selection acting on it. Mean juvenile body mass at the onset of winter has declined continuously over the last two decades in this population (from 15.69 kg in 1997 to 12.98 kg in 2014), with the steadily earlier onset of spring over time [39]. Phenotypic variance in juvenile body mass at Chizé is mainly driven by cohort effects and by differences in maternal environment that influence maternal provisioning and thereby offspring growth. This suggests that selection is acting mainly on the environmental part of the trait. In other words, among-individual genetic variation in body mass did not offset the effects of a deteriorating environment. By contrast, in the Bogesund population, where no detectable viability selection occurred on neonatal body mass, we observed a substantial level of genetic variance and heritability for the same trait. This study highlights the importance of considering the impact of spatial (i.e. between populations) and temporal (i.e. among cohorts) heterogeneity in environmental conditions, especially during early life stages, for understanding the genetic architecture of the potential responses of wild populations to environmental changes. Our findings also demonstrate the importance of assessing the evolutionary potential of critical traits for fitness in populations faced with contrasting environmental contexts so as to predict the effect of a given environmental change on population persistence in a conservation perspective [21].

## Additional file

Additional file 1: Pedigree-reconstruction method (Supp text 1) and analysis of viability selection on neonatal body mass (Supp Text 2, Figure S1). (DOCX 16292 kb)

## Abbreviations

CV: Coefficient of Variation
JBM: Juvenile Body Mass
LRT: Likelihood Ratio Test
MA: Maternal Additive genetic
ME: Maternal permanent Environmental
