# Supplementary material for: Between-population differences in the genetic and maternal components of body mass in roe deer
Source: BMC Evol Biol. 2018 Mar 28;18:39. doi: 10.1186/s12862-018-1154-9 (PMC5872551; doi:10.1186/s12862-018-1154-9)
Supplement: Supplementary file 1 — Pedigree-reconstruction method (Supp text 1) and analysis of viability selection on neonatal body mass (Supp Text 2, Figure S1). (DOCX 16292 kb) [file 12862_2018_1154_MOESM1_ESM.docx]

**Supplementary material**

**Supplementary text 1. Pedigree reconstruction**

The maximum likelihood parentage-assignment method in COLONY2 allows simultaneous estimation of maternity, paternity, full-sibships and paternal or maternal half-sibships. It implements a clustering method whereby individuals are placed into family groups with the highest likelihood. Since statistical power increases with sibship size, this method is particularly appropriate for species such as roe deer which may have two or even three offspring per year. To enhance this benefit, we ran analyses on offspring belonging to three successive birth cohorts. The whole study period was covered using a “sliding-window” approach, with windows of three years/cohorts and an overlap of two years. Individuals of known age were thus included in three successive analyses (e.g. individuals born in 1998 were analysed in three runs: 1996-1998, 1997-1999 and 1998-2000). Offspring first captured as adults (35 % in CH, 50% in BOG) were included in 5 to 9 successive analyses depending on the margins of error for estimating their age (+1 for individuals estimated as 2 year-old, ±1 for individuals estimated as 3 year old, ±2 for individuals estimated as 3-6 years-old deer and ± 3 for individuals estimated as older than 6 years-old, Hewison et al. 1999).

Based on long-term capture-mark-recapture, hunting databases, and yearly field observations, we listed the candidate fathers and mothers for each cohort. Both males and females were considered as candidate parents for a given fawn cohort if they were recorded alive and potentially reproducing during the previous rut. The last potential participation in the rut for an individual was defined in relation to the time it was either found dead, or considered dead based on annual re-capture rates (i.e. when the probability of being alive but not caught dropped below 1%). The first potential participation in the rut was set to 1 year-old for females and to 2 years old for males in relation to the age of sexual maturity in roe deer. Although male roe deer become territorial late in their third winter, some non-territorial 2-year-old males may participate in the rut (Liberg et al. 1998) and successfully breed (Vanpé et al. 2009). Again, when the age of a candidate parent was estimated from tooth wear, the first year of potential participation in the rut was back-dated in order to take into account potential error in age estimation (using the margins of error detailed above). Adults of unknown age (i.e. N = 194 individuals caught at CH between 2009 and 2012 with no age estimate) were considered to be present and participate in the rut up to 4 years prior to their first capture based on captures rates. However, they were not analyzed as offspring. We retained inferences with a level of confidence > 95% and which were fully congruent in at least three runs.

**Supplementary text 2. *Assessing viability selection on neonatal body mass***

We tested for the existence of detectable viability selection on neonatal body mass by quantifying the response of early fawn survival to neonatal body mass in the two populations. We expected a strong viability selection on early body mass at Chizé, but not at Bogesund where fawn rearing conditions are much more favorable [38]

*Material and Methods*

To test for viability selection on neonatal body mass at Bogesund and Chizé, we assessed the influence of neonatal mass on survival the first winter using a logistic regression implemented in the “stats” R package (R Development Core Team 2016). At Bogesund, fawns were equipped with very high frequency (VHF) radio-collars and were regularly (once or twice a week) located by radio-telemetry and triangulation from marking until 180 days after birth. The radio-collars were equipped with a mortality sensor, providing information on the current fate of each fawn. At Chizé, fawns marked as neonates during spring were considered to have survived their first summer/autumn if they were captured in a subsequent winter capture (i.e. at 8 months of age, 20 months, and so on). Indeed, the high recapture probability means that the chance of surviving fawns escaping capture for several years is very low [32, 35]. We used data for 216 fawns (born between 1997 and 2005) in Bogesund and 319 fawns (born between 1995 and 2012) in Chizé. We fitted a population by mass interaction to test formally for a difference in viability selection in both populations.

*Results*

The magnitude of viability selection on neonatal body mass markedly differed between populations (*χ^2^*=8.54, d.f. = 1, P=0.003). We found strong evidence of viability selection at Chizé (mean coefficient ± SE = 0.46 ± 0.15, P<0.001) (Figure S1). A neonate weighing 1kg had a predicted survival probability of 0.27, whereas a 3 kg neonate had a predicted survival probability of 0.48. In contrast, we did not find any effect of mass on fawn survival in Bogesund (mean coefficient ± SE = -0.23 ± 0.19, P=0.22). The same result was obtained when adjusting neonate body mass by capture age (only available at Bogesund).

**Figure S1. Relationship between the probability of surviving (measured over the first 8 months of life) and fawn body mass (measured within the first 3 weeks**) at Chizé (in red) and Bogesund (in blue). The lines represent model predictions of the logistic regression with their 95% CIs. Points (which are proportional in size to the sample size) indicate the average survival probabilities for each class of 1kg mass.

*
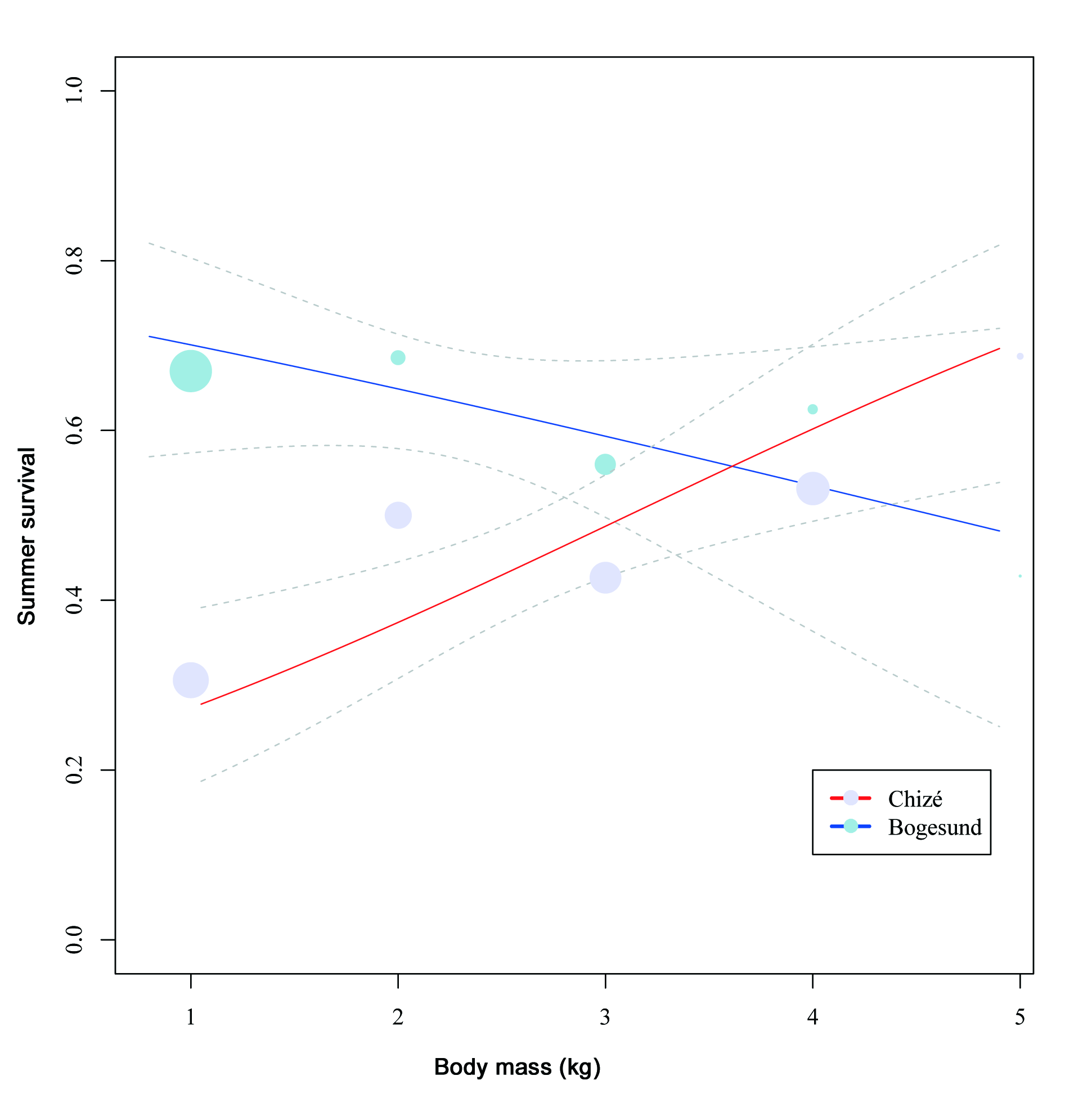
*
